# Novel approach using serum progesterone as a triage to guide management of patients with threatened miscarriage: a prospective cohort study

**DOI:** 10.1038/s41598-020-66155-x

**Published:** 2020-06-04

**Authors:** Thiam Chye Tan, Chee Wai Ku, Lee Koon Kwek, Kai Wei Lee, Xiaoxuan Zhang, John C. Allen, Valencia Ru-Yan Zhang, Nguan Soon Tan

**Affiliations:** 1 Department of Obstetrics and Gynaecology, KK Women’s and Children’s Hospital, 100 Bukit Timah Road, 229899 Singapore, Singapore; 2 Duke-National University of Singapore Medical School, 8 College Road, 169857 Singapore, Singapore; 3 Lee Kong Chian School of Medicine, Nanyang Technological University Singapore, 11 Mandalay Road, 308232 Singapore, Singapore; 4 Centre for Quantitative Medicine, Duke-National University of Singapore Medical School, Singapore, 20 College Road, Academia, 169856 Singapore; 5 Yong Loo Lin School of Medicine, National University of Singapore, NUHS Tower Block Level 11, 1E Kent Ridge Road, 119228 Singapore, Singapore; 6 School of Biological Sciences, Nanyang Technological University Singapore, 60 Nanyang Drive, 637551 Singapore, Singapore

**Keywords:** Chemical biology, Health care, Medical research, Risk factors, Biomarkers

## Abstract

Threatened miscarriage is a common gynaecological emergency, with up to 25% of women eventually progressing to spontaneous miscarriage. The uncertainty of pregnancy outcomes results in significant anxiety. However, there is currently no acceptable framework for triaging patients presenting with threatened miscarriage. We aim to evaluate the efficacy and safety of a novel clinical protocol using a single serum progesterone level to prognosticate and guide management of patients with threatened miscarriage. 1087 women presenting with threatened miscarriage were enrolled in the study. The primary outcome was spontaneous miscarriage by 16 weeks’ gestation. Among the 77.9% (847/1087) of study participants with serum progesterone ≥ 35 nmol/L who were not treated with oral dydrogesterone, the miscarriage rate was 9.6% (81/847). This did not differ significantly from the 8.5% (31/364) miscarriage rate observed in our prior studies; *p* = 0.566. Among women with serum progesterone < 35 nmol/L who were treated with dydrogesterone, the miscarriage rate was 70.8% (170/240). Our novel clinical triage protocol using a single serum progesterone level allowed both effective risk stratification and a reduction in progestogen use with no significant adverse pregnancy outcomes. This protocol, based on a single serum progesterone cutoff, can be readily adapted for use in other healthcare institutions.

## Introduction

Threatened miscarriage—defined as per vaginal bleeding with or without abdominal pain in early pregnancy, is the most common gynaecological emergency, occurring in 15–20% of all pregnancies^1^ with 20–25% eventually progressing to spontaneous miscarriage^2^. Women with threatened miscarriage have been reported to experience significant anxiety and depressive symptoms due to their uncertain pregnancy outcomes^3^. Lack of a clinical protocol to effectively prognosticate and triage these women, compounded by inconclusive evidence regarding progestogen use in the management of threatened miscarriage, further complicates the situation.

The use of progestogens in managing threatened miscarriage has always been controversial. In the recently published PRISM trial in women with no history of previous miscarriage, treatment with progesterone in women experiencing bleeding during the first 12 weeks of pregnancy did not result in a significant increase in the incidence of live births compared to treatment with placebo^4^. On the other hand, limited systematic reviews and meta-analyses^1,5–7^ have shown a reduction in miscarriage risk when women with threatened miscarriage were treated with progestogens. However, the validity of these studies may be questioned owing to small sample sizes and methodological weaknesses^1^. At present, according to the latest NICE guidelines, conservative management is recommended for threatened miscarriage^8^.

Prior to implementing the spot serum progesterone triage, all women presenting with threatened miscarriage at our institution were routinely prescribed oral progestogens — which is also the common practice in many other countries. Although there have been no serious maternal adverse events or adverse fetal outcomes reported thus far from the use of progestogens in threatened miscarriage^1^, there is a possible association between progestogen use and development of birth defects^9^, hypospadias^10^ and congenital heart disease in the offspring^11^. In addition, progestogen use may be associated with adverse effects such as nausea, vomiting^5^ and breast fullness^12^. Hence, a review of the current practice surrounding progestogen use is warranted in an effort to reduce such risks.

We have previously shown that serum progesterone is lower in women presenting with threatened miscarriage compared to those with low risk pregnancies^13^. In particular, women with spontaneous miscarriage exhibited even lower serum progesterone that did not increase with gestation length. On the other hand, women with higher progesterone levels were found to have lower risk of miscarriage. Thus, serum progesterone may be a useful serum biomarker for predicting outcomes in patients with threatened miscarriage^14^. Serum progesterone was used as a screening tool for triaging risk of miscarriage amongst women who presented with threatened miscarriage in a pilot study conducted at our institution between 2012 to 2015. The cut-off point for serum progesterone based on the study was 35 nmol/L^15^, and this result was subsequently validated in a much larger cohort^16^. These studies demonstrated that serum progesterone levels of ≥ 35 nmol/L had a 92% negative predictive value for excluding subsequent miscarriage.

In this study, we aimed to evaluate efficacy and safety of a novel clinical protocol that uses a spot serum progesterone level to triage and guide subsequent management of patients presenting with threatened miscarriage. The protocol was implemented in KK Women’s and Children’s Hospital from January 2017 to December 2018 in women presenting with threatened miscarriage at the emergency department.

## Materials and Methods

### Study design

This prospective, single centre cohort study was conducted from 1 January 2017 to 31 December 2018 at KK Women’s and Children’s Hospital (KKH), the largest maternity hospital in Singapore. This study was reviewed and approved by the SingHealth Centralized Institutional Review Board (CIRB) of Singapore (Reference number 2017/2638). Informed consent was obtained from all individual participants enrolled into the study. All research described in this manuscript was performed in accordance with relevant guidelines and regulations.

### Study participants

Study participants were women presenting with threatened miscarriage at the KKH emergency department, the Urgent O&G Clinic (UOGC). Women meeting the pre-determined inclusion criteria (Supplementary Fig. S1) were recruited into the study. The inclusion criteria specified a single intrauterine pregnancy between weeks 5 to 12 of gestation confirmed and dated via ultrasonography. For women in whom ultrasonography was not performed, number of days since the last menstrual period (LMP) was used to calculate gestational age. Exclusion criteria included progestogen treatment during the current pregnancy, heavy bleeding with a Pictorial Blood Loss Assessment Chart (PBAC) score of >1, multiple gestations, incomplete or inevitable miscarriage, pregnancy of unknown location or *in-vitro* fertilization pregnancy.

### Serum measurements

Serum progesterone level was determined from maternal blood samples at presentation. Blood was collected in plain tubes and centrifuged for 10 minutes at 3000 *g* within 2 hours of collection. Serum progesterone level was subsequently measured in the KKH clinical laboratory using a commercial ARCHITECT progesterone kit (Abbott, Ireland).

### Maternal characteristics

Information on maternal demographics and obstetric factors was collected via an investigator-administered questionnaire in either English or Chinese.

### Clinical protocol

Women with serum progesterone < 35 nmol/L were stratified into the “high-risk” group and treated with oral dydrogesterone as per the manufacturer’s protocol (Duphaston, Abbott), given anticipatory guidance and monitored closely. Women with serum progesterone levels ≥ 35 nmol/L were stratified into the “low-risk” group and conservatively managed via counselling and reassurance with no progestogen treatment. All study participants were reviewed in a KKH clinic 2 weeks later and followed up until week 16 of gestation to ascertain pregnancy outcome.

### Outcomes measured

The primary outcome was spontaneous miscarriage, defined as self-reported uterine evacuation after an inevitable or incomplete miscarriage, or complete miscarriage with an empty uterus by week 16 of gestation. Pregnancy outcome was determined via a phone call to study participants at week 16 of gestation and clinically confirmed to verify pregnancy status.

### Statistical methods

Statistical analysis was performed using SAS version 9.4 (SAS Institute Inc., Cary, NC). Baseline maternal demographics and pregnancy characteristics were statistically compared between the study cohorts. A 2-sample t-test was used to compare continuous baseline variables and Fisher’s exact test was used to compare categorical variables. Univariate analyses were employed to assess progesterone levels and other maternal factors on risk of spontaneous miscarriage and multivariate logistic regression was subsequently performed to account for any potential confounders. To account for missing BMI data in the study population, multiple imputation (10 simulations) by fully conditional specification (FCS) was performed.

### Details of ethics approval

This study was reviewed and approved by the SingHealth Centralized Institutional Review Board (CIRB) of Singapore (Reference number 2017/2638).

## Results

**Figure 1:**
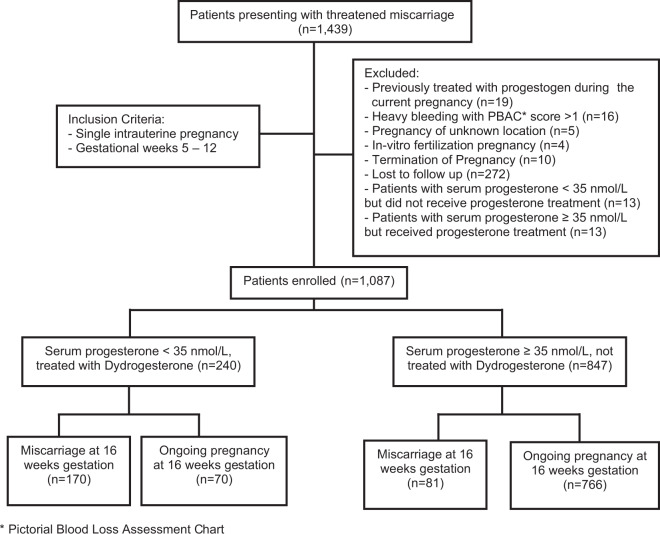
Clinical outcomes of patients presenting with threatened miscarriage and triaged using serum progesterone. *Pictorial Blood Loss Assessment Chart.

1,439 women presented with threatened miscarriage during the study period. A total of 1,087 women met the pre-specified inclusion criteria and were subsequently included for analysis (Fig. 1).

77.9% (8471087) of patients had serum progesterone ≥ 35 nmol/L (low-risk group) and 22.1% (240/1087) had serum progesterone < 35 nmol/L (high-risk group). Comparison of baseline characteristics showed a higher body mass index (BMI) (26.5 vs 24.9 kg/m^2^, *p* = 0.006) in the high-risk group of women. Even though a higher maternal age (31.8 vs 30.8 years, *p* = 0.017) and lower gestational age (6.8 vs 7.3 weeks, *p* = 0.002) were also reported in this group of women, these differences were not clinically significant.

23.1% (251/1087) of study patients experienced spontaneous miscarriage prior to 16 weeks of gestation. Despite receiving treatment with progestogens, 70.8% (170/240) of patients with serum progesterone levels < 35 nmol/L and 9.6% (81/847) of those with serum progesterone levels ≥ 35 nmol/L experienced spontaneous miscarriage (Table 1).

**Table 1:** Baseline characteristics, mean serum progesterone and pregnancy outcomes of patients presenting with threatened miscarriage.

Characteristic	Serum Progesterone < 35 nmol/L (n = 240)	Serum Progesterone ≥ 35 nmol/L (n = 847)	P-value
Maternal Age (years)	31.8 (31.1–32.5)	30.8 (30.5–31.1)	0.017
Body Mass Index, BMI (kg/m^2^)	26.5 (25.4–27.6)	24.9 (24.4–25.4)	0.006
Gestational Age (weeks)	6.8 (6.7–7.0)	7.3 (7.2–7.5)	0.002
**Mean serum progesterone (nmol/L)**			
All study participants	20.8 (19.8–21.8)	65.1 (63.6–66.6)	<0.001
Patients who miscarried	18.0 (16.9–19.1)*	59.4 (55.6–63.2)^†^	
Patients with ongoing pregnancy	27.5 (26.2–28.9)*	65.7 (64.0–67.3)^†^	
**Pregnancy Outcome**			
Number of patients who miscarried	170 (70.8%)	81 (9.6%)	<0.001
Number of patients with ongoing pregnancy	70 (29.2%)	766 (90.4%)	

Data presented as mean (95% CI) or n (%).

*Within group analysis for serum progesterone < 35 nmol/L: Mean serum progesterone for patients who miscarried vs. patients with ongoing pregnancy (18.0 nmol/L vs. 27.5 nmol/L, ρ < 0.001).

^†^Within group analysis for serum progesterone ≥ 35 nmol/L: Mean serum progesterone for patients who miscarried vs. patients with ongoing pregnancy (59.4 nmol/L vs. 65.7 nmol/L, ρ = 0.028).

In a comparison with our pilot^15^ and validation^16^ cohorts in which all women were given oral progestogen treatment, withholding progestogen treatment in women with serum progesterone ≥ 35 nmol/L in the present study resulted in a 78% reduction in progestogen use with no significant increase in the miscarriage rate. In the present study, the miscarriage rate was 9.6% (81/847), whereas in the combined pilot and validation cohorts, the miscarriage rate was 8.5% (31/364) (*p* = 0.566) (Supplementary Table S1).

**Figure 2:**
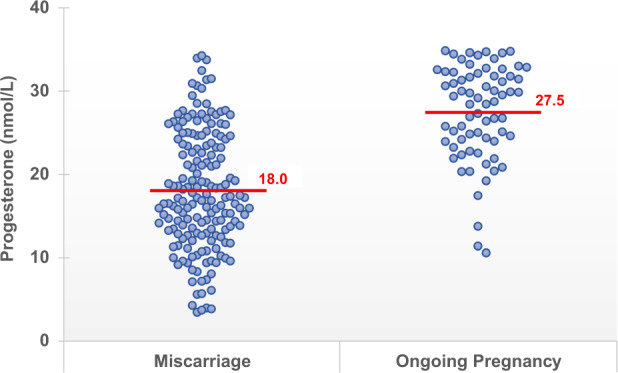
Subgroup analysis of patients with serum progesterone < 35 nmol/L – Serum progesterone distribution in patients with spontaneous miscarriage at 16 weeks gestation compared with patients with ongoing pregnancy at 16 weeks gestation.

A subgroup analysis in women with serum progesterone < 35 nmol/L showed that mean serum progesterone amongst those who miscarried was significantly lower than those with ongoing pregnancy (18.0 nmol/L vs. 27.5 nmol/L, *p* < 0.001) (Table 1 and Fig. 2).

Univariate and multivariate logistic regression analysis identified serum progesterone and maternal age as significant risk factors for miscarriage, taking into account the influence of BMI and gestational age. Higher serum progesterone was shown to reduce the risk of spontaneous miscarriage across both (< 35 nmol/L, ≥ 35 nmol/L) groups. Specifically, every unit increase in serum progesterone was associated with a 21% reduction (on average) in risk (odds) of miscarriage for women in the high-risk (< 35 nmol/L) group. In the low-risk (≥ 35 nmol/L) group, a higher gestational age at presentation appeared to be the most significant factor in reducing the risk of miscarriage, with every unit increase associated with a 27% reduction in risk. Although serum progesterone was also a significant factor influencing risk of miscarriage in the low-risk group of women, it contributed only a 3% increase in miscarriage risk for every unit increase. Across both groups, higher maternal age was also found to increase the risk of spontaneous miscarriage (Table 2).

**Table 2:** Multivariate logistic regression analysis of factors associated with miscarriage.

Factor	Serum Progesterone <35 nmol/L		Serum Progesterone ≥35 nmol/L	
	Odds Ratio (95% CI)	P-value	Odds Ratio (95% CI)	P-value
Serum Progesterone	0.80 (0.75–0.85)	<0.001	0.976 (0.96–0.99)	0.003
Maternal Age	1.092 (1.02–1.17)	0.016	1.124 (1.06–1.19)	<0.001
BMI	0.93 (0.84–1.02)	0.119	0.91 (0.83–1.00)	0.054
Gestational Age	0.921 (0.706–1.201)	0.544	0.732 (0.60–0.90)	0.003

13 women with progesterone levels < 35 nmol/L declined treatment with oral progestogens, and they were excluded from the analysis. However, it was interesting to note that all of these women miscarried.

## Discussion

By using a novel approach that incorporates spot serum progesterone measurement at presentation to triage women with threatened miscarriage and guide subsequent management, this study provides early evidence of an effective and safe clinical protocol (Supplementary Fig. S1).

Efficacy of the clinical protocol is demonstrated by the accuracy in predicting pregnancy outcomes at 16 weeks of gestation amongst the two risk-stratified groups. The majority of women in the low-risk group (serum progesterone ≥ 35 nmol/L) had an ongoing pregnancy (90.4%) while the majority of women in the high-risk group (serum progesterone < 35 nmol/L) had a spontaneous miscarriage despite progestogen treatment (70.8%) (Table 1).

In a comparison with our pilot^15^ and validation^16^ cohorts in which all women were given oral progestogen treatment, withholding progestogen treatment in women with serum progesterone ≥ 35 nmol/L did not result in a significant increase in the miscarriage rate in this subgroup of women (9.6% vs 8.5%, *p* = 0.566) (Supplementary Table S1). This demonstrates the safety of the new protocol with a 77.9% reduction in progestogens use without increasing the risk of miscarriage. In line with the principle of parsimony, withholding treatment would also mean a reduction in adverse outcomes and side effects associated with all medical therapy, a reduction in use of limited hospital resources and also increased financial savings for the patient. Nonetheless, adequate anticipatory guidance would be essential because 9.6% of the low-risk group still experienced spontaneous miscarriage at 16 weeks of gestation.

For the 13 high-risk group of women with progesterone levels < 35 nmol/L who declined progestogen treatment, all of them miscarried. On the other hand, 70.8% of the high-risk group of women still miscarried eventually, even after treatment with oral progestogens. This might be attributed to other proposed aetiologies of early miscarriage regardless of serum progesterone levels, including chromosomal abnormalities, infections and maternal disease states such as autoimmune conditions^17^.

The strength of this study is that it is the largest prospective cohort study of a novel clinical protocol, using a validated serum progesterone cut-off to triage women presenting with threatened miscarriage, to guide management and treatment based on the risk of miscarriage. This paves the way for future validation in other cohorts and clinical settings with a relative ease of implementation involving only a single blood test.

A limitation of the study is that the presence of a fetal pole or fetal cardiac activity was not factored into the outcome of the pregnancies. It has been shown that the presence of fetal cardiac activity is a favourable prognostic factor in pregnancies with threatened miscarriage^18^. However, detection of a fetal pole and/or cardiac activity is dependent on the gestational age of the foetus and many of our patients presented at an early gestation. Amongst those with low serum progesterone, the mean gestational age was lower compared to those with a high serum progesterone. This could possibly account for some women at early gestation without fetal cardiac activity, who go on to a spontaneous miscarriage. Thus, the objective measurement of serum progesterone levels, especially in pregnancies of unknown viability, remains even more useful in risk stratification, without having to wait for the development of a positive fetal heart before adequate anticipatory guidance can be provided. This allows women with high serum progesterone to be reassured, and further treatment withheld, without significant increase in the incidence of miscarriage in this group of women.

Incorporating this novel triage protocol with serum progesterone lends further weight to our earlier work on the pivotal role of progesterone in early pregnancy development^13,15,16^. Furthermore, higher serum progesterone is shown to be protective against miscarriage in the high-risk group of women with serum progesterone < 35 nmol/L. Progesterone is an essential hormone in pregnancy. It sustains decidualization, controls uterine contractility and promotes maternal immune tolerance to the fetal semi-allograft. Luteal phase deficiency (LPD), which is caused by a delay in transition between corpus luteum derived progesterone and placental derived progesterone, is proposed to be a major factor in early miscarriages^19^. In patients with luteal phase defects, such as in those undergoing assisted reproduction, progesterone supplementation is well-documented to improve pregnancy rates^20,21^. In our current study, amongst women with low serum progesterone of < 35 nmol/L, 29.2% were successfully treated with oral progestogens with an ongoing pregnancy at 16 weeks of gestation. Interestingly, the reported rates of LPD were much lower, between 4–9%, in healthy women of reproductive age^22–24^. The incidence of LPD in the population of women with threatened miscarriage and low serum progesterone may therefore be higher.

Apart from LPD, other proposed causes of early miscarriage include chromosomal abnormalities, infections, and maternal disease states such as autoimmune conditions like antiphospholipid syndrome and systemic lupus erythematosus^12^. These may account for spontaneous miscarriage in women regardless of serum progesterone levels. This should form the basis of future studies on early spontaneous miscarriage, because we have yet to find both an answer to the aetiology, or a cure to prevent spontaneous miscarriage in this population. Understanding the underlying pathophysiology behind the miscarriage may lead to novel targets, both for prediction and treatment of this group of women. However, underlying biological heterogeneity as evidenced by a different serum progesterone level but the same clinical phenotype of miscarriage may make this task extremely daunting.

In addition, future randomized controlled trials should include a trial of progesterone treatment versus placebo for treatment of high-risk women with threatened miscarriage and serum progesterone < 35 nmol/L. A defective signalling pathway downstream of progesterone may contribute to treatment failure. This would allow closer monitoring of women who are more likely to experience bleeding with a subsequent miscarriage despite oral progestogen treatment.

## Conclusion

Overall, our study demonstrated that a safe and effective clinical protocol using spot serum progesterone level in women presenting with threatened miscarriage allowed accurate risk stratification as well as reduction in the use of oral progestogen treatment without significant difference in miscarriage rate. In the high-risk cohort of women (serum progesterone < 35 nmol/L), higher serum progesterone was protective against miscarriage. This has far-reaching clinical implications, because it establishes a safe clinical protocol that can be readily adapted for use in other healthcare institutions with only a single serum progesterone test. Patients with high serum progesterone levels can be reassured and counselled without medical treatment, while patients with low serum progesterone levels have high risk of miscarriage even with treatment.

## Supplementary information

Supplementary Figure S1

Supplementary Table S1

## References

[1] Wahabi HA, Fayed AA, Esmaeil SA, Bahkali KH (2018). Progestogen for treating threatened miscarriage. Cochrane Database Syst Rev.

[2] Kouk LJ (2013). A prospective study of risk factors for first trimester miscarriage in Asian women with threatened miscarriage. Singapore Med J.

[3] Zhu CS (2018). Threatened miscarriage and depressive and anxiety symptoms among women and partners in early pregnancy. J Affect Disord.

[4] Coomarasamy A (2019). A Randomized Trial of Progesterone in Women with Bleeding in Early Pregnancy. N Engl J Med.

[5] Carp H (2012). A systematic review of dydrogesterone for the treatment of threatened miscarriage. Gynecol Endocrinol.

[6] Lee HJ, Park TC, Kim JH, Norwitz E, Lee B (2017). The Influence of Oral Dydrogesterone and Vaginal Progesterone on Threatened Abortion: A Systematic Review and Meta-Analysis. Biomed Res Int.

[7] Mirza FG, Patki A, Pexman-Fieth C (2016). Dydrogesterone use in early pregnancy. Gynecol Endocrinol.

[8] National Collaborating Centre for, W. S. & Children’s, H. In *Ectopic Pregnancy and Miscarriage: Diagnosis and Initial Management in Early Pregnancy of Ectopic Pregnancy and Miscarriage* (Rcog National Collaborating Centre for Women’s and Children’s Health., 2012).

[9] Queisser-Luft A (2009). Dydrogesterone use during pregnancy: overview of birth defects reported since 1977. Early Hum Dev.

[10] Huang Y, Wang HY, Li PQ, Xing P (2017). [Risk factors for different types of hypospadias]. Zhonghua Nan Ke Xue.

[11] Zaqout M (2015). The Impact of Oral Intake of Dydrogesterone on Fetal Heart Development During Early Pregnancy. Pediatr Cardiol.

[12] Saharkhiz N (2016). A comparative study of dydrogesterone and micronized progesterone for luteal phase support during *in vitro* fertilization (IVF) cycles. Gynecol Endocrinol.

[13] Ku CW (2018). Serum progesterone distribution in normal pregnancies compared to pregnancies complicated by threatened miscarriage from 5 to 13 weeks gestation: a prospective cohort study. BMC Pregnancy Childbirth.

[14] Arck PC (2008). Early risk factors for miscarriage: a prospective cohort study in pregnant women. Reprod Biomed Online.

[15] Ku CW (2015). How can we better predict the risk of spontaneous miscarriage among women experiencing threatened miscarriage?. Gynecol Endocrinol.

[16] Lek SM (2017). Validation of serum progesterone <35 nmol/L as a predictor of miscarriage among women with threatened miscarriage. BMC Pregnancy Childbirth.

[17] Andrews MA (2015). Dietary factors and luteal phase deficiency in healthy eumenorrheic women. Hum Reprod.

[18] Sotiriadis A, Papatheodorou S, Makrydimas G (2004). Threatened miscarriage: evaluation and management. Bmj.

[19] Schindler AE (2004). First trimester endocrinology: consequences for diagnosis and treatment of pregnancy failure. Gynecol Endocrinol.

[20] Fatemi HM (2009). The luteal phase after 3 decades of IVF: what do we know?. Reprod Biomed Online.

[21] van der Linden, M., Buckingham, K., Farquhar, C., Kremer, J. A. & Metwally, M. Luteal phase support for assisted reproduction cycles. *Cochrane Database Syst Rev*, Cd009154, 10.1002/14651858.CD009154.pub3 (2015). 10.1002/14651858.CD009154.pub3 PMC6461197 26148507

[22] Lenton EA, Landgren BM, Sexton L (1984). Normal variation in the length of the luteal phase of the menstrual cycle: identification of the short luteal phase. Br J Obstet Gynaecol.

[23] Schliep KC (2014). Luteal phase deficiency in regularly menstruating women: prevalence and overlap in identification based on clinical and biochemical diagnostic criteria. J Clin Endocrinol Metab.

[24] Smith SK, Lenton EA, Landgren BM, Cooke ID (1985). Is the short luteal phase a defective luteal phase?. Ann N Y Acad Sci.

