# Supplementary material for: Novel approach using serum progesterone as a triage to guide management of patients with threatened miscarriage: a prospective cohort study
Source: Sci Rep. 2020 Jun 4;10:9153. doi: 10.1038/s41598-020-66155-x (PMC7272626; doi:10.1038/s41598-020-66155-x)

**Supplementary Figure S1**. Clinical workflow implemented in KKH Emergency Department using spot serum progesterone as a triage to guide management of women presenting with threatened miscarriage


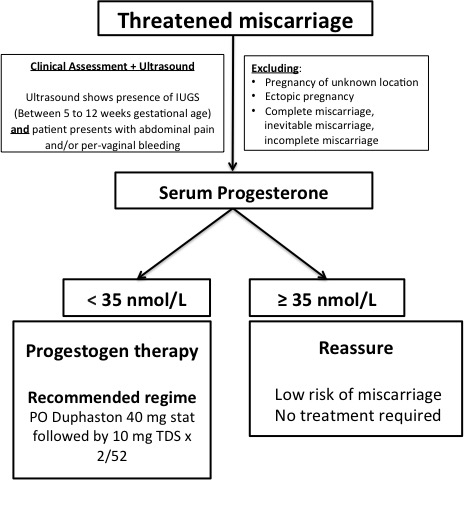

Supplement: Supplementary file 1 — Supplementary Figure S1 [file 41598_2020_66155_MOESM1_ESM.docx]
