# Supplementary material for: Novel approach using serum progesterone as a triage to guide management of patients with threatened miscarriage: a prospective cohort study
Source: Sci Rep. 2020 Jun 4;10:9153. doi: 10.1038/s41598-020-66155-x (PMC7272626; doi:10.1038/s41598-020-66155-x)
Supplement: Supplementary file 2 — Supplementary Table S1 [file 41598_2020_66155_MOESM2_ESM.docx]

**Supplementary Table S1.** Comparison of miscarriage rates between current study cohort with

that from the pilot and validation cohorts

| **Characteristic** | **Current Study Cohort** | **Pilot and Validation Cohorts** | **P-value** |
| --- | --- | --- | --- |
| All pregnancies | n = 1,087 | n = 465 |  |
| Patients who miscarried | 251 (23.1%) | 100 (21.5%) | 0.493 |
| Patients with ongoing pregnancy | 836 (76.9%) | 365 (78.5%) |  |
| Pregnancies with serum progesterone ≥ 35 nmol/L | n = 847 | n = 364 |  |
| Patients who miscarried | 81 (9.6%) | 31 (8.5%) | 0.566 |
| Patients with ongoing pregnancy | 766 (90.4%) | 333 (91.5%) |  |

Data are presented as n (%)
